# *Fusobacterium nucleatum* confers chemoresistance by modulating autophagy in oesophageal squamous cell carcinoma

**DOI:** 10.1038/s41416-020-01198-5

**Published:** 2020-12-10

**Authors:** Yang Liu, Yoshifumi Baba, Takatsugu Ishimoto, Hiroyasu Tsutsuki, Tianli Zhang, Daichi Nomoto, Kazuo Okadome, Kensuke Yamamura, Kazuto Harada, Kojiro Eto, Yukiharu Hiyoshi, Masaaki Iwatsuki, Yohei Nagai, Shiro Iwagami, Yuji Miyamoto, Naoya Yoshida, Yoshihiro Komohara, Masaki Ohmuraya, Xiaoming Wang, Jaffer A. Ajani, Tomohiro Sawa, Hideo Baba

**Affiliations:** 1 Second Oncology Department, Shengjing Hospital affiliated of China Medical University, Shenyang, Liaoning China; 2 Department of Gastroenterological Surgery, Graduate School of Medical Sciences, Kumamoto University, Kumamoto, Japan; 3 Department of Next-Generation Surgical Therapy Development, Graduate School of Medical Sciences, Kumamoto University, Kumamoto, Japan; 4 International Research Center for Medical Sciences, Kumamoto University, Kumamoto, Japan; 5 Department of Microbiology, Graduate School of Medical Sciences, Kumamoto University, Kumamoto, Japan; 6 Department of Cell Pathology, Graduate School of Medical Sciences, Kumamoto University, Kumamoto, Japan; 7 Department of Genetics, Hyogo College of Medicine, Hyogo, Japan; 8 Radiology Department, Shengjing Hospital affiliated of China Medical University, Shenyang, Liaoning China; 9 Department of Gastrointestinal Medical Oncology, The University of Texas MD Anderson Cancer Center, Houston, TX USA; 10 Center for Metabolic Regulation of Healthy Aging, Kumamoto University, Kumamoto, Japan

**Keywords:** Tumour biomarkers, Oesophageal cancer

## Abstract

**Background:**

*Fusobacterium nucleatum* (*F. nucleatum*) is a gut microbe implicated in gastrointestinal tumorigenesis. Predicting the chemotherapeutic response is critical to developing personalised therapeutic strategies for oesophageal cancer patients. The present study investigated the relationship between *F. nucleatum* and chemotherapeutic resistance in oesophageal squamous cell carcinoma (ESCC).

**Methods:**

We examined the relationship between *F. nucleatum* and chemotherapy response in 120 ESCC resected specimens and 30 pre-treatment biopsy specimens. In vitro studies using ESCC cell lines and co-culture assays further uncovered the mechanism underlying chemotherapeutic resistance.

**Results:**

ESCC patients with *F. nucleatum* infection displayed lesser chemotherapeutic response. The infiltration and subsistence of *F. nucleatum* in the ESCC cells were observed by transmission electron microscopy and laser scanning confocal microscopy. We also observed that *F. nucleatum* modulates the endogenous LC3 and ATG7 expression, as well as autophagosome formation to induce chemoresistance against 5-FU, CDDP, and Docetaxel. ATG7 knockdown resulted in reversal of *F. nucleatum*-induced chemoresistance. In addition, immunohistochemical studies confirmed the correlation between *F. nucleatum* infection and ATG7 expression in 284 ESCC specimens.

**Conclusions:**

*F. nucleatum* confers chemoresistance to ESCC cells by modulating autophagy. These findings suggest that targeting *F. nucleatum*, during chemotherapy, could result in variable therapeutic outcomes for ESCC patients.

## Background

Oesophageal cancer is one of the leading causes of cancer-related death worldwide. Squamous cell carcinoma is the most common pathological type of oesophageal cancer in the world, especially in Asia, parts of Africa, and Europe.^1,2^ The prognosis of oesophageal cancer is poor and only about 19% of the patients survive for 5 years.^3^ Based on multiple clinical studies, preoperative chemotherapy or chemoradiation followed by esophagectomy has become the preferred multimodal treatment for patients with resectable oesophageal cancer.^4,5^ Docetaxel, cisplatin (CDDP), and 5-fluorouracil (FU) are the key chemotherapeutic agents used to treat oesophageal squamous cell carcinoma (ESCC).^6,7^ Identification of novel therapeutic targets in ESCC could potentially improve the risk-adapted treatment strategies and help develop rational clinical trials, in which patients may be selected based on biomarkers.

In recent years, there has been an increasing interest in the relationship between the gut microbiome and cancer. Previous studies confirmed that gut microbiome is closely related to chemotherapy and immunotherapy responses in cancer patients.^8–11^ Increasing evidence suggest that *Fusobacterium nucleatum*, a species in the gut microbiota, is linked to cancer*. F. nucleatum* is a Gram-negative, non-sporulating anaerobic bacterium^12^ found in the human oral and gastrointestinal tract.^13^ *F. nucleatum* not only causes oral diseases, such as periodontitis and gingivitis, but is also closely related to the occurrence and development of gastrointestinal cancers.^12,14^ A previous study revealed that the adhesive and invasive abilities of *F. nucleatum* to epithelial cells might be one of the possible causes promoting tumour metastases.^15^ Numerous studies have reported that the abundance of *F. nucleatum* correlated with poor prognosis in patients with gastrointestinal cancer, especially colorectal cancer (CRC),^16^ further supporting its role in imparting aggressive tumour phenotype. We previously reported that *F. nucleatum* is associated with shorter survival and inferior chemotherapeutic response in oesophageal cancer, suggesting its potential role as a prognostic or predictive biomarker.^17,18^ Interestingly, a recent study revealed that *F. nucleatum* promotes chemoresistance in CRC by modulating autophagy.^19^ Hence, we hypothesised that *F. nucleatum* might confer chemotherapeutic resistance via autophagy in ESCC.

To test this hypothesis, we examined the relationship between *F. nucleatum* and poor chemotherapy response in 120 resected ESCC specimens and 30 pre-treatment biopsy specimens. In addition, our experimental study clarified that *F. nucleatum* infiltrates and survives inside ESCC cells and induces autophagy, leading to the acquisition of resistance to chemotherapeutic agents like Docetaxel, 5-FU, and CDDP. Our results might potentially help in designing targeted therapy for *F. nucleatum*-induced chemoresistance in ESCC patients to improve their prognosis.

## Methods

### Patients

To examine the correlation between *F. nucleatum* and the chemotherapeutic response in ESCC patients, we utilised cohort A including 120 patients who underwent surgical resection at Kumamoto University Hospital between 2005 and 2017. All patients in cohort A had preoperative (neoadjuvant) chemotherapy (CDDP and 5-FU either with or without Docetaxel). Tumour staging was performed according to the American Joint Committee on Cancer Staging Manual (Seventh edition).^20^ Patient characteristics of cohort A are summarised in Table 1. Positron emission tomography (PET) data were available for 95 patients of cohort A; their characteristics are summarised in Supplementary Table 1. Next, we analysed pre-treatment biopsy specimens from 30 ESCC patients (cohort B) who received chemotherapy (CDDP, 5-FU, and Docetaxel) between 2011 and 2014. Cohort B included 17 resectable tumours (stage II+III) and 13 unresectable tumours (stage IV). The characteristics of patients from cohort B are summarised in Supplementary Table 2. Third, cohort C was utilised to evaluate the relationship between *F. nucleatum* and autophagy in clinical specimens. Cohort C included 284 ESCC patients who underwent surgical resection from 2010 to 2016. This cohort included both patients with preoperative treatment and those without preoperative treatment. Patient characteristics of Cohort C are summarised in Supplementary Table 3. We previously reported a relationship between *F. nucleatum* and chemotherapy response;^18^ however, compared with that work, the present study utilised a larger number of resected cases (*n* = 120; cohort A), 30 newly examined pre-treatment specimens (cohort B), and investigated the relationship between *F. nucleatum* and autophagy (*n* = 284; cohort C). Written informed consent was obtained from each patient and the institutional review boards of all the participating institutions approved this study (#1364).

**Table 1:** Clinicopathological features (cohort A).

	*F. nucleatum* expression		*P* value
	Low (*n* = 85)	High (*n* = 35)	
Mean age ± SD	67 (41–86)	65 (48–89)	0.78
Sex			0.69
Male	73	31	
Female	12	4	
Location			0.83
Upper	59	25	
Lower	26	10	
T stage			0.014
T1	13	3	
T2	16	1	
T3, T4	56	31	
N stage			0.78
N0	6	3	
N1–3	79	32	
Chemotherapy regimen			0.78
Docetaxel + CDDP + 5-FU	80	30	
CDDP + 5-FU	5	5	

### Chemotherapeutic response evaluation

**Fig. 1:**
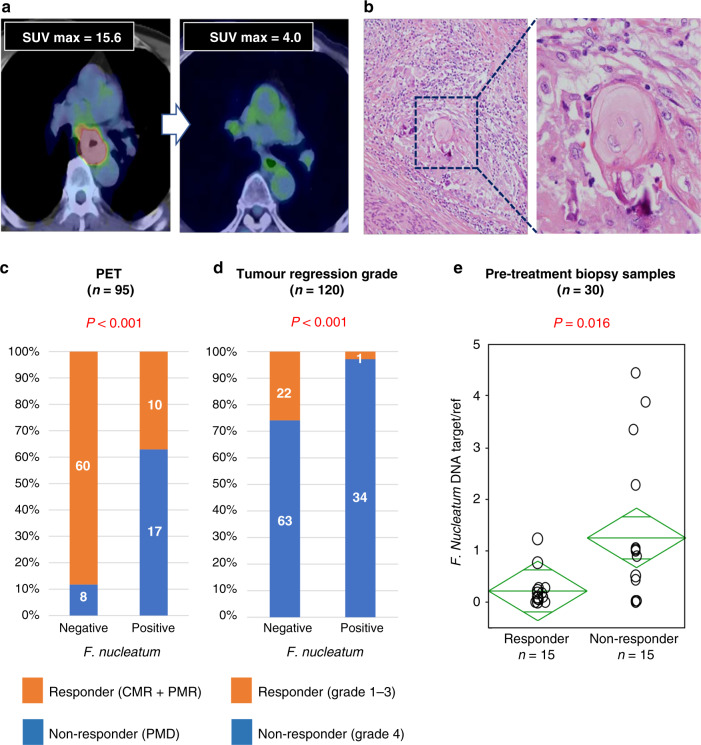
*F. nucleatum* and chemotherapy response in clinical samples. Representative findings (**a** CT, PET, **b** pathology) in ESCC patients with favourable therapeutic effect. **c**, **d** Chemotherapeutic response rate according to *F. nucleatum* status using **c** SUV max response and **d** tumour regression grade. **e** The relationship between *F. nucleatum* DNA amount in pre-treatment biopsy specimens and RECIST.

First, 95 out of 120 patients (cohort A) underwent co-registered PET/computed tomography (CT) using a hybrid PET/CT imager (Gemini GXL16, Philips Medical Solutions, Amsterdam, The Netherlands). Standardised uptake value (SUV) response was classified as follows:^21^ complete metabolic response (CMR)—complete resolution of fluorodeoxyglucose (FDG) uptake within the measurable target lesion with the appearance of no new lesion; partial metabolic response (PMR)—at least 30% reduction in SUV max of FDG uptake; progressive metabolic disease (PMD)—>30% increase in the SUV max of the FDG uptake or the appearance of FDG avid new lesion/s that is/are typical morphology of cancer; stable metabolic disease (SMD)—disease that did not qualify for CMR, PMR, or PMD. Patients with CMR or PMR were defined as responders (Fig. 1a). Second, the histopathological response to neoadjuvant chemotherapy was classified into four categories according to the following criteria:^22^ grade 1, no evidence of viable tumour cells; grade 2, <10% viable tumour cells; grade 3, 11–50% viable tumour cells; and grade 4, >50% viable tumour cells. Subsequently, grade 1–3 tumours were combined with a responding group, while grade 4 tumours were classified as a non-responding group (Fig. 1b).

### Immunohistochemical staining

The slides were incubated with the primary antibodies (1:100 dilution, rabbit monoclonal antibody (mAb) for ATG7, ab 52472, Abcam, UK; 1:500 dilution, rabbit mAb for LC3, 3868 CST, USA; 1:200 dilution, rabbit mAb for Beclin-1, 3395 CST, USA; and 1:75 dilution, ki-67, clone MIB-1, Dako, Santa Clara, CA, US) overnight at 4 °C and with Anti-rabbit Envision+/horseradish peroxidase (HRP) (Dako) or Anti-mouse Envision+/HRP (Dako) secondary antibody for 1 h. Staining of ATG-7, LC3, and Beclin-1 was categorised semi-quantitatively based on the percentage of positive tumour cells: 0 (5% positive cells), 1 (6–25% positive cells), 2 (26–50% positive cells), 3 (51–75% positive cells), and 4 (>75% positive cells). Cytoplasmic and membrane staining intensities were also determined semi-quantitatively as follows: 0 (negative), 1 (weakly positive), 2 (moderately positive), and 3 (strongly positive). Sections’ scores were defined as the extent of staining × intensity, while the Ki-67 score was calculated as the percentage of positively stained cells among the total number of malignant cells scored. The scoring was conducted using three high-power (×400) fields in the invasive edge of the tumour, which represents the spectrum of staining observed in the whole section.

### Detection of *F*. nucleatum DNA using quantitative real-time PCR (qPCR)

*F. nucleatum* DNA levels were measured from the formalin-fixed paraffin-embedded tissues of ESCC patients as previously reported by Mima et al.^23^ The amount of *F. nucleatum* DNA in each sample was normalised to human *SLCO2A1*.

### Culture of *F. nucleatum*

*F. nucleatum* was obtained from Riken Bioresource Center Microbe division (No. 8532, Japan). Under sterile conditions, freeze-dried strain was dissolved in liquid culture medium [Gifu Anaerobic Medium (GAM) broth; Nissui Pharmaceutical Co., Tokyo, Japan]. The culture was incubated at 37 °C for 24 h under anaerobic condition by using AnaeroPack-Anaero (Mitsubishi Gas Chemical Company Inc., Tokyo, Japan) and stored at −80 °C in 25% glycerol. The frozen stock solution was inoculated to a bacterial culture tube containing 5 ml of GAM broth. To generate the third-generation working species, 100 μl of the second-generation bacterial solution was added to 5 ml of GAM broth and cultured for 6 h. After calculation of bacterial colony-forming units (CFU) by measuring optical density at 600 nm (OD600), the bacteria were washed with 5 ml of phosphate-buffered saline (PBS) using high-speed centrifugation at 10,000 rpm for 3 min (3×) and then used for co-culture experiments.

### Cell lines

Human oesophageal cancer cell lines (TE 8 and TE-10) were obtained from the Cell Resource Center for Biomedical Research, the Riken BioResource Center Cell Bank, and the Japanese Collection of Research Bioresources Cell Bank. The cell lines had been tested and authenticated by Cell ID System in October 2018 (TE8) and June 2019 (TE10).

### Cell proliferation and apoptosis assessment

Tumour cell proliferation was monitored using IncuCyte (Essen BioScience, USA); cellular growth curve was presented as the confluent cell percent. The drug-treated cells were seeded in 96-well plates at a density of 5000 per well and cultured in IncuCyte ZOOM for monitoring. For apoptosis, IncuCyte^@^Annexin V green reagent (4642, Essen BioScience, USA) were added into the wells simultaneously along with the drug. Each well was imaged with 4 independent fields using a ×10 objective, in an interval of 3 h. Apoptosis was measured by the total green object area in the acquired images.

### Transmission electron microscopy (TEM)

The samples were fixed in 2% glutaraldehyde (GA) and 4% paraformaldehyde (PFA) at room temperature for 30 min and washed thrice with 0.1 M cacodylate buffer. After washes, the cells were collected and centrifuged at 15,000 rpm for 5 min. 50 μl of 10% albumin was added to the cell pellet and 2.5% GA was used as the overlay for incubation at 4 °C overnight. With the increasing concentration gradient of ethanol for dehydration, the samples were embedded at 60 °C in the oven for 48 h. The samples were then cut into sections and stained with lead citrate and observed using a TEM (HT7700, HITACHI, Japan).

### Invasion assay

Invasion of *F. nucleatum* in TE8 and TE10 was quantified by antibiotic protection assay as mentioned elsewhere.^24,25^ Briefly, TE8 and TE10 cells were seeded in a 96-well flat-bottom culture plate at a density of 5 × 10^4^ cells per well. Before co-culture with *F. nucleatum*, the cells were washed with PBS and cultured in antibiotic-free RPMI media. *F. nucleatum* was inoculated into GAM broth and cultured under anaerobic condition. After calculating the CFU per millilitre by measuring OD600, *F. nucleatum* was washed with PBS and resuspended in RPMI. *F. nucleatum* was added to the cells at a multiplicity of infection (MOI) of 100 and were incubated at 37 °C under 5% CO_2_ for 4 h. After incubation, the unattached bacteria were removed by washing three times with PBS. Fresh medium containing 200 μg/ml metronidazole and 300 μg/ml gentamicin was added to eliminate extracellular bacteria and incubated for 1 h. Our in vitro experiment results showed that this concentration of antibiotics completely kills 5 × 10^7^/ml *F. nucleatum* within 1 h. After exposure to the antibiotics, the cells were washed and lysed with 200 μl of sterile distilled water for 10 min at 37 °C. Serial dilutions of the lysates with PBS were plated on GAM agar plates and incubated anaerobically at 37 °C for 54 h. The viable bacteria count was determined by CFU counting on the plates. Each experiment was performed in triplicate and repeated at least twice.

### Laser scanning confocal microscopy (LSCM) of *F. nucleatum*

ESCC cells were seeded in a glass bottom dish and allowed to reach 50–70% confluence, after which *F. nucleatum* was co-cultured for 4 h. After washed with PBS 2 times, the cell membrane was stained using wheat germ agglutinin, Alexa Fluor ^@^647 conjugate (W32466, Thermo Fisher Scientific, USA) for 5 min in the incubator. The cells were then fixed using 4% PFA for 10 min and permeabilised using 0.1% Triton X-100 for 1 min. After the incubation of the sample with the primary antibody (1:200 dilution of rabbit Anti-*F. nucleatum*, ANT0084, DIATHEVA, Italy) overnight at 4 °C, Alexa FluorTM 488 goat anti-rabbit Ig(H + L)(A11008, Thermo Fisher Scientific, USA) was used as the secondary antibody and incubated for 1 h in the dark. The sample was then counterstained with 4,6-diamidino-2-phenylindole (DAPI; KT013, DOJINTO, Japan). The samples were observed under confocal laser scanning microscope (FV1200, OLYMPUS, Japan).

### LSCM of endogenous LC3

ESCC cells were seeded in a glass bottom dish and allowed to reach 50–70% confluence after which it was co-cultured with *F. nucleatum* for 4 h. After washing with PBS twice, the cells were fixed using 4% PFA for 10 min and permeabilised with 0.1% Triton x-100 for 1 min. All samples were then incubated with the primary rabbit Anti-*F. nucleatum* antibody (1:200 dilution, ANT0084, DIATHEVA, Italy) and mouse mAb LC3 (1:100 dilution, CTB-LC3–2-IC, COSMO BIO CO. LTD., Japan) overnight at 4 °C. Alexa FluorTM 488 goat anti-rabbit Ig(H + L) (A11008, Thermo Fisher Scientific, USA) and Alexa Fluor 594 mouse (A11005, Thermo Fisher Scientific, USA) were used as secondary antibodies and incubated for 1 h in the dark. DAPI (KT013, DOJINTO, Japan) was used to counterstain the nucleus. The observation was performed under confocal laser scanning microscope (FV1200, OLYMPUS, Japan) or the samples were preserved at 4 °C.

### Statistical analysis

All the experiments were repeated three times independently. The data management and analysis were performed using GraphPad Prism 7.00. All *P* values were two tailed, and *t* test was used to compare the two groups. *P* < 0.05 was considered significant.

## Results

### *F. nucleatum* and chemotherapy response in clinical samples

We assessed the relative amounts of *F. nucleatum* DNA in ESCC tissues by qPCR assay. *F. nucleatum* was detected in 35 (29%) of 120 cases. We first probed for any significant changes in chemotherapy response in patients positive for *F. nucleatum*. The metabolic response rates determined by SUVmax values were obtained from PET/CT imaging. The analyses revealed that patients with a higher burden of *F. nucleatum* had significantly fewer responders (i.e. patients with CMR or PMR; 37.0% (10/27) vs. 88.2% (60/68) in the low *F. nucleatum* group; *P* < 0.001; Fig. 1c). We also performed the pathological assessment of all patients based on the tumour regression grade (TRG) analysis. In these analyses, we noted that *F. nucleatum* levels were significantly higher in ESCC patients with a poor vs. better pathological response to chemotherapy (TRG 4 vs. TRG 1, 2 and 3; *P* < 0.001; Fig. 1d). To evaluate the effect of tumour stage, we examined the relationship between *F. nucleatum* and chemotherapeutic response among T3 patients only and found similar results (Supplementary Fig. A). Finally, utilising 30 biopsy samples, we observed a significant relationship between *F. nucleatum* DNA amount in pre-treatment specimens and chemotherapeutic response (*P* = 0.016; Fig. 1e). Taken together, these results illustrate that patients with high intratumoural levels of *F. nucleatum* appear to have greater resistance to chemotherapeutic treatment.

### *F. nucleatum* invades and survives in ESCC cells

**Fig. 2:**
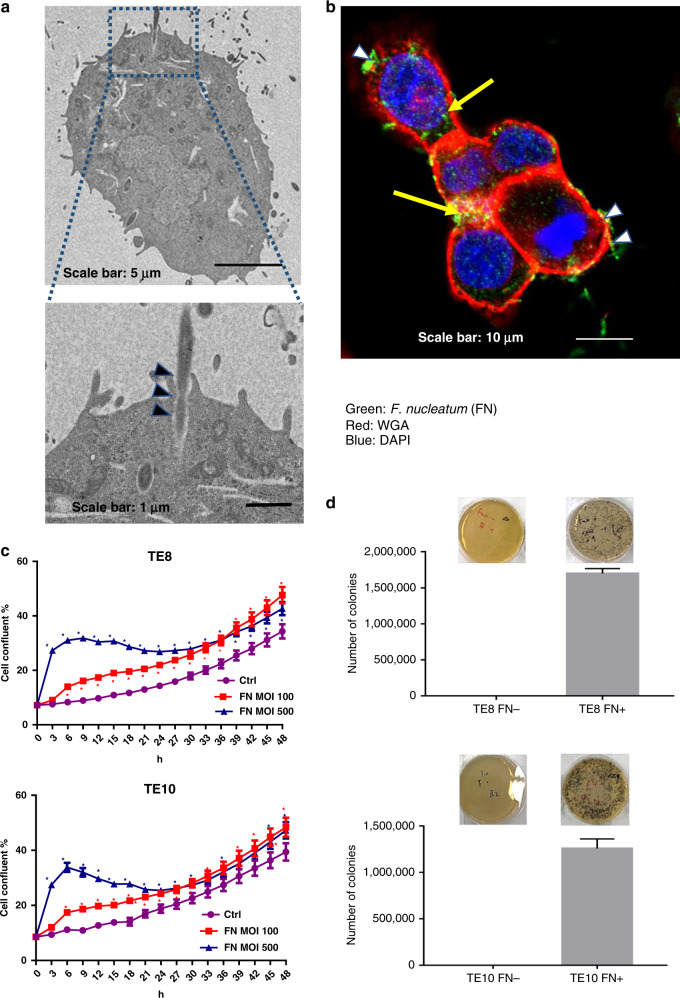
*F. nucleatum* invades and survives in ESCC cells. **a** *F. nucleatum* invades into the cancer cell as observed by transmission electron microscopy (17,500 ×3 magnification) in TE10 cell. Scale bar, 1 µm. **b** LSCM analysis of *F. nucleatum* adhered and invading into the TE10 cells. Cytomembrane (red), cell nucleus (blue), and *F. nucleatum* (green). Black arrowheads indicate invasion in **a**, and white arrowheads indicate adherence in **b**. **c** Proliferation assay by Incucyte, with or without *F. nucleatum*, for 48 h at MOI 100 and 500 in TE8 cells and TE10 cells. *P < 0.05 (red and blue) indicates significant difference between the MOI 100 and ctrl groups and between the MOI 500 and ctrl groups. **d** *F. nucleatum* invasion in TE8 and TE10 cancer cell lines. Growth of intracellular *F. nucleatum* into colonies on the GAM agar.

Using multiplex visualisation methods such as TEM and LSCM, we observed the dynamic state of *F. nucleatum* and ESCC cell lines in the co-culture assay. In addition, the TEM analysis demonstrated the penetration of long rod shape *F. nucleatum* into the plasma membrane of ESCC cells (Fig. 2a). The LSCM analysis also confirmed the presence of the fluorescent-labelled *F. nucleatum* inside ESCC cells (Fig. 2b). The growth assay was used to assess cell proliferation under co-culture condition. *F. nucleatum* induced ESCC cell proliferation under a multiplicity of infection (MOI) of 100 and 500 (Fig. 2c). To assess the ability of invasion and survival of *F. nucleatum* in the cells, we performed the minimum inhibitory concentration assay. The results demonstrated that *F. nucleatum* (5 × 10^7^/ml) was eliminated by treating the cells with metronidazole (200 μg/ml) and gentamicin (300 μg/ml) for 1 h (Supplementary Fig. B). The ESCC cells were co-cultured with *F. nucleatum* (MOI = 100) for 4 h. To eliminate the extracellular *F. nucleatum*, metronidazole (200 μg/ml) and gentamicin (300 μg/ml) were added and incubated for 1 h. Notably, we observed that the cell lysate formed colonies on the agar after 54 h under anaerobic conditions (Fig. 2d). No colony formation was observed in ESCC cells without *F. nucleatum*. The most striking result from these data is that *F. nucleatum* not only has the ability of adhesion and invasion but also survive as an intracellular pathogen, which might be a reason for the enrichment of *F. nucleatum* in ESCC tissues.

### *F. nucleatum* and autophagy in ESCC cells

**Fig. 3:**
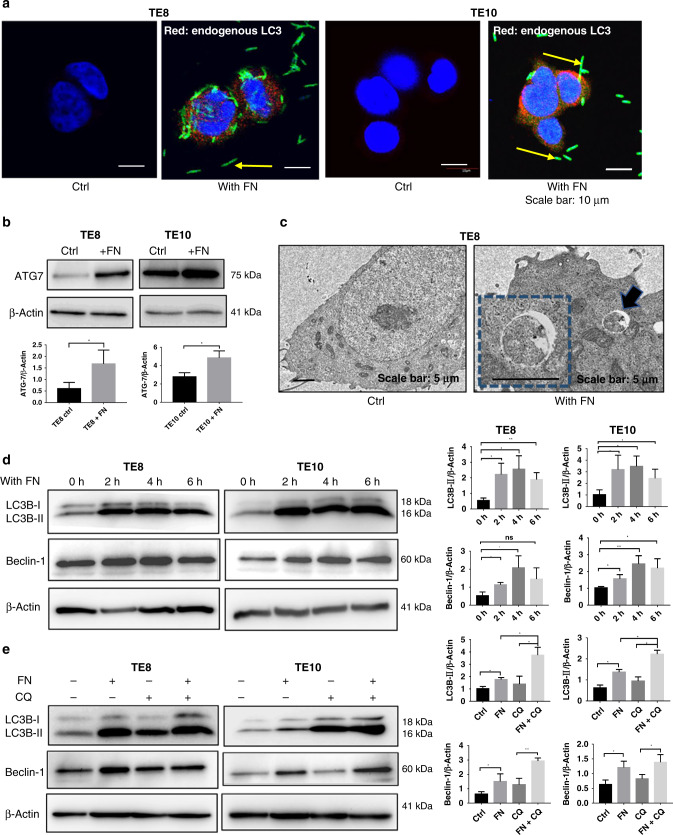
*F. nucleatum* and autophagy in ESCC cells. **a** Endogenous LC3 is upregulated by *F. nucleatum* in TE8 and TE10 cells. LSCM images depicting LC3 (red), *F. nucleatum* (Green), and the cell nucleus (blue). **b** Western blot analysis of ATG7 in TE8 cells and TE10 cells co-cultured with *F. nucleatum*. Summary graphs show quantification of western blot image. Statistical significance is denoted by *P < 0.05. **c** Autophagosomes in TE8 cells cultured with *F. nucleatum* as observed by transmission electron microscopy (17,500 ×3 magnification). Scale bar, 1 µm. **d** Western blot analysis of LC3B-II and Beclin-1 in TE8 cells and TE10 cells co-cultured with *F. nucleatum*. Summary graphs show quantification of the western blot image. Statistical significances are denoted by *P < 0.05, **P < 0.01, ns not significant. **e** Western blot analysis of LC3B-II and Beclin-1 in TE8 cells and TE10 cells co-cultured with *F. nucleatum* in the presence of chloroquine. Summary graphs show quantification of the western blot image. Statistical significances are denoted by *P < 0.05, **P < 0.01.

Detecting LC3 by immunoblotting or immunofluorescence is the most reliable method for monitoring autophagy.^26,27^ In the present study, we performed immunofluorescent staining for LC3 to detect the endogenous LC3 with or without *F. nucleatum*. Compared with the ESCC cells without *F. nucleatum*, the endogenous LC3 was observed predominantly punctate in the cytoplasm by LSCM in *F. nucleatum* co-culture groups (Fig. 3a). ATG7 is considered to be essential molecules for the induction of autophagy; *ATG-7* gene encodes an E1-like activating enzyme and participates in autophagosome formation in the canonical pathway and hence crucial for autophagy and cytoplasmic to vacuole transport.^28^ Compared to the control group, ATG7 expression increased significantly in the *F. nucleatum* co-cultured group both in TE8 and TE10 cells, which are evident from the western blot analyses (Fig. 3b). As the cytoplasmic contents are sequestered by the autophagosome, a double-membrane structure, during autophagy, we next observed the autophagosomes in TE8 cells using TEM. Autophagosome was rarely seen in the cytoplasm of TE8 cells. However, autophagosome was observed in these cells co-cultured with *F. nucleatum* (Fig. 3c). LC3B-II protein was used as a marker for autophagy^29^ and mammalian Beclin-1, another autophagy-related marker, is a homolog of the yeast Atg6/vacuole protein sorting 30 (Vps30) gene.^30^ We observed that both LC3B-II and Beclin-1 were upregulated in the *F. nucleatum* co-culture groups after co-cultured for 2, 4, and 6 h (Fig. 3d). Furthermore, the autophagy inhibitor, chloroquine (CQ) was used to intervene autophagy in TE8 cells and TE10 cells co-cultured with *F. nucleatum*. Western blot results showed higher expression of LC3B-II and Beclin-1 in *F. nucleatum* with CQ treatment than the control. Further, the accumulation of LC3B-II in *F. nucleatum* co-cultured group was higher than the control group suggesting that *F. nucleatum* might promote autophagy in TE8 and TE10 cells (Fig. 3e). Collectively, these results provide important insights into the induction of autophagy by *F. nucleatum* in ESCC.

### *F. nucleatum* and chemotherapy resistance in ESCC cells

**Fig. 4:**
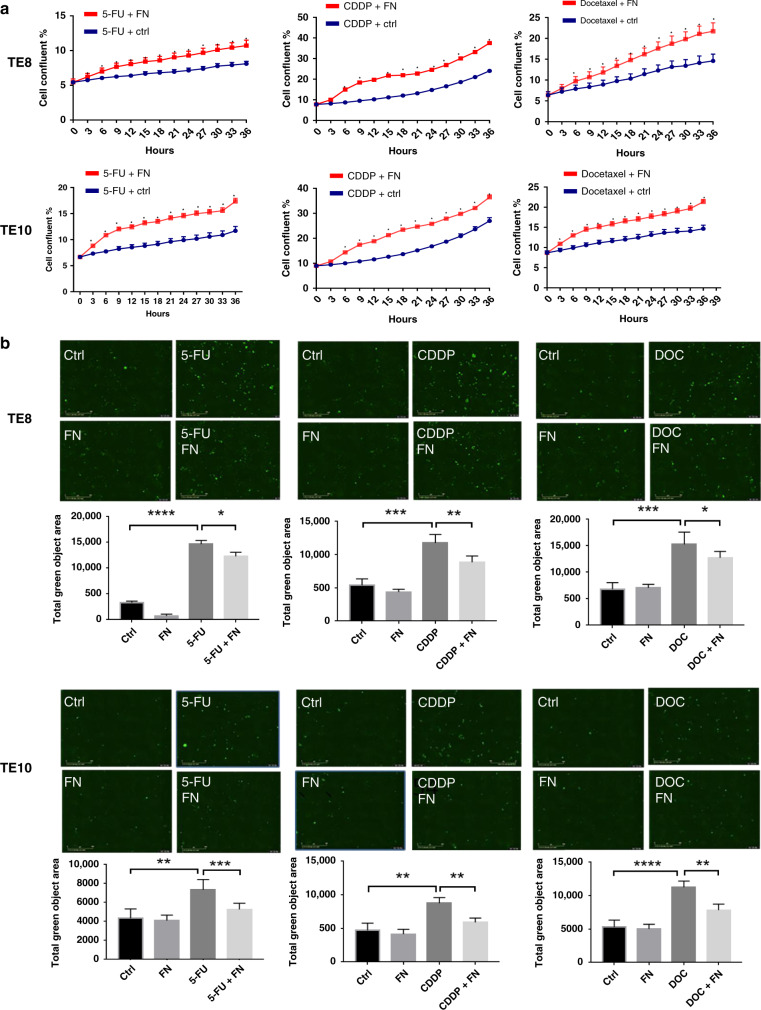

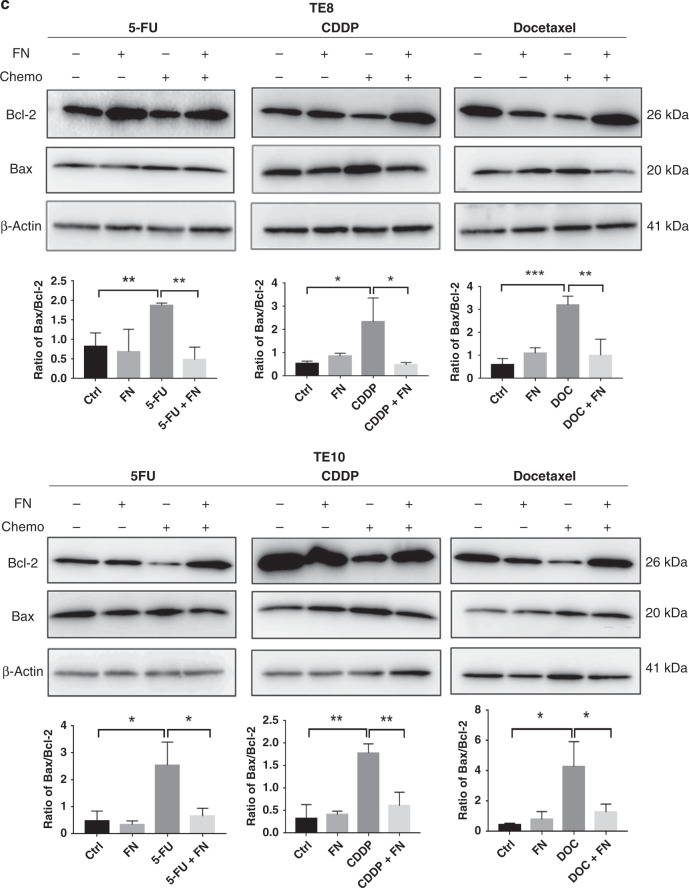
*F. nucleatum* and chemotherapy resistance in ESCC cells. **a** Proliferation assay in TE8 and TE10 cells with or without *F. nucleatum* treated with 5-FU, CDDP, and Docetaxel. Statistical significance is denoted by *P < 0.05. **b** Apoptosis assay using Annexin V green at 12 h with or without *F. nucleatum* under 5-FU, CDDP, and Docetaxel treatment in TE8 and TE10 cells. The total green object area indicates the apoptosis index. Statistical significance is denoted by **P < 0.01, ***P < 0.001, and ****P < 0.0001. **c** Western blot analysis to check the expression of Bcl-2 and Bax in TE8 and TE10 cells upon treatment with 5-FU, CDDP, and Docetaxel in the presence or absence of *F. nucleatum*. Statistical significance is denoted by *P < 0.05, **P < 0.01, ***P < 0.001.

First, we measured the IC50 of the chemotherapeutic agents 5-FU, CDDP, and Docetaxel on TE8 and TE10 cell lines (Supplementary Fig. C). The chemotherapeutic agents 5-FU, CDDP, and Docetaxel were added to the *F. nucleatum* (MOI = 100) co-cultured TE8 cells and TE10 cells. Cell proliferation assay showed that the growth rate of TE8 and TE10 cells of *F. nucleatum* co-cultured group was significantly higher than the control group, suggesting that *F. nucleatum* induces resistance to chemotherapy in ESCC cells (Fig. 4a). To clarify whether apoptosis is induced in ESCC cells upon co-culture with *F. nucleatum* under chemotherapeutic condition, we performed apoptosis assay to detect the phosphatidylserine externalisation using Annexin V reagent. The results showed that the apoptosis induced by the chemotherapeutic agents was significantly decreased in *F. nucleatum* co-cultured TE8 and TE10 (Fig. 4b). The crucial apoptotic proteins pro-apoptotic Bax and anti-apoptotic Bcl-2 are widely used to evaluate cellular apoptosis. The ratio of Bax/Bcl-2 is a reliable marker for apoptosis detection.^31^ The results indicated a reduction in the ratio of Bax/Bcl-2 further confirming that *F. nucleatum* inhibits apoptosis in ESCC cells upon 5-FU, CDDP, and Docetaxel exposure (Fig. 4c).

### *F. nucleatum* confers chemoresistance by modulating autophagy in ESCC

**Fig. 5:**
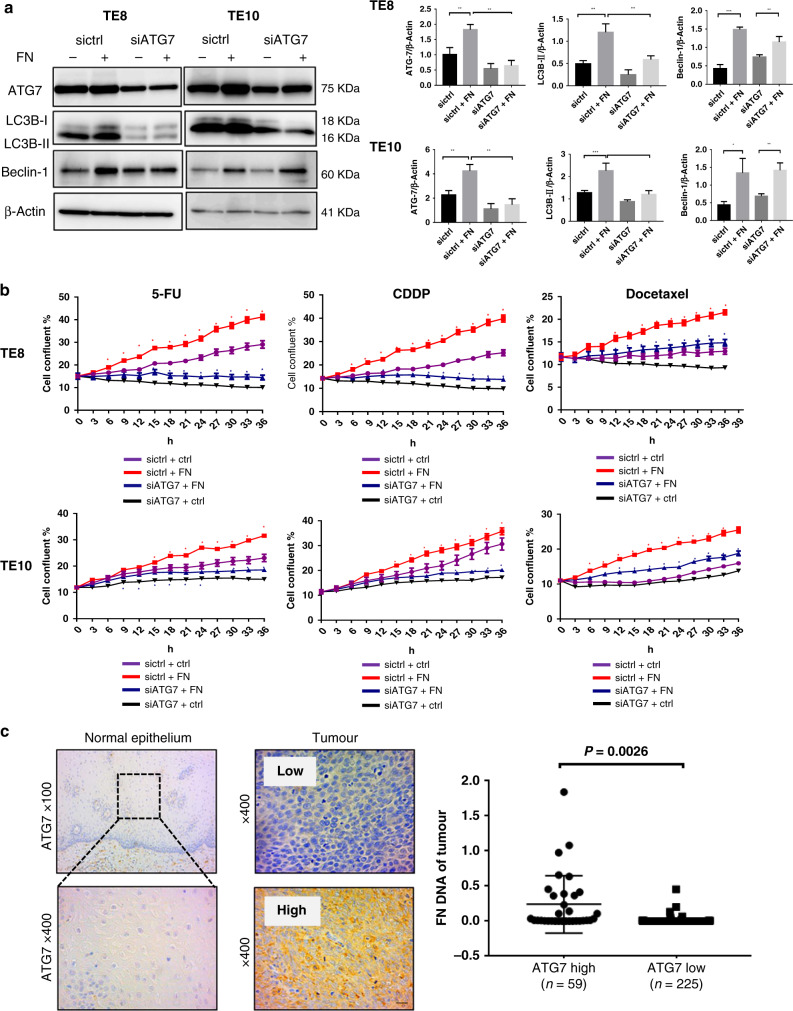
*F. nucleatum* confers chemoresistance by modulating autophagy in ESCC. **a** Western blot analysis of ATG7, LC3B and Beclin-1 expression in TE8 and TE10 cells transfected with siATG7 and co-cultured with *F. nucleatum*. Statistical significances are denoted by *P < 0.05, **P < 0.01, ***P < 0.001. **b** Proliferation assay in siATG7-transfected TE8 cells and TE10 cells co-cultured with *F. nucleatum* and treated with 5-FU, CDDP, and Docetaxel. *P < 0.05 (red and blue) indicates significant difference between the sictrl + ctrl and sictrl + FN groups and the siATG7 + ctrl and siATG7 + FN groups. **c** Immunohistochemical analysis of ATG7 protein expression in normal epithelial tissue and tumour lesions of ESCC patients. Higher *F. nucleatum* DNA was observed in the ATG7 high expression group (*n* = 59) compared to the low expression group (*n* = 225) (*P* = 0.0026).

To confirm that autophagy plays a pivotal role in the chemoresistance induced by *F. nucleatum*, we used two small interfering RNA (siRNA) against ATG7. The efficiency of ATG7 knockdown was confirmed by qPCR analysis in TE8 and TE10 cells (Supplementary Fig. D). The protein expression of LC3B-II, ATG7, and Beclin-1 in siATG7-transfected TE8 cells and TE10 cells, co-cultured with and without *F. nucleatum*, were investigated by western blotting analysis. In *F. nucleatum* co-cultured groups, siATG7 blocked the *F. nucleatum*-induced LC3 cleavage, suggesting that *F. nucleatum* might activate autophagy cascade by upregulating ATG7 expression in the host cells. (Fig. 5a), whereas Beclin-1 levels remained unchanged upon ATG7 knockdown. However, its expression was upregulated when the cells were co-cultured with *F. nucleatum*, irrespective of ATG7 status. Additionally, the growth rate upon *F. nucleatum* infection in 5-FU-, CDDP-, and Docetaxel-treated TE8 and TE10 cells transfected with siATG7 was assessed. The growth rate of *F. nucleatum*-infected, ATG7 knocked down cells were significantly lower than that of the control group. CQ could decrease the growth of TE8 and TE10 in *F. nucleatum* group compared with that in only *F. nucleatum* group, indicating that CQ could increase the sensitivity of *F. nucleatum* co-cultured cells to chemotherapeutic drugs (Supplementary Fig. E). The most striking result is that *F. nucleatum* induces proliferation in ESCC cells, while ATG7 knockdown reversed the *F. nucleatum*-induced chemoresistance in ESCC (Fig. 5b). To ensure that the findings are indeed not due to off-target effects, we performed these experiments with another siRNA targeting ATG7 and observed similar results (data not shown). These results support that *F. nucleatum* confers chemoresistance by modulating autophagy.

The relative amounts of *F. nucleatum* DNA were quantified using qPCR and ATG7 expression was evaluated by immunohistochemistry analysis in cohort C with 284 ESCC patients’ specimens. We observed significantly higher levels of *F. nucleatum* DNA in the ATG7 high group (59/284, 20.8%) than the ATG7 low group (225/284, 79.2%) (*P* = 0.0026; Fig. 5c). Moreover, we conducted immunohistochemical analysis of the autophagy markers LC3 and Beclin-1. The expression levels of LC3 (*P* = 0.015) and Beclin-1 (*P* = 0.024) were significantly higher in *F. nucleatum*-positive tumours than in *F. nucleatum*-negative tumours (Supplementary Fig. F). Taken together, these results may indicate a positive correlation between the expression of autophagy markers and *F. nucleatum* DNA in samples from ESCC patients. In addition, the immunohistochemical expression of Ki-67, a proliferation index, was higher in samples from ATG7 high *F. nucleatum* (+) patients compared with those from ATG7 low *F. nucleatum* (−) patients (*P* = 0.013) in cohort C (Supplementary Fig. G), which implies the potential of *F. nucleatum* DNA and ATG7 expression levels as viable clinical markers of disease progression.

Finally, antibiotics (gentamicin and metronidazole) could reverse the proliferation of ESCC with *F. nucleatum* in chemotherapeutic drugs (5-FU, CDDP, and Docetaxel) (Supplementary Fig. H).

## Discussion

In the current scenario, preoperative chemotherapy or chemoradiation followed by esophagectomy is the preferred multimodal treatment for patients with resectable ESCC. Hence, the development of a predictive biomarker for these ESCC patients is increasingly important. There are various methods to assess the therapeutic response during cancer treatment. Complementary anatomical and pathological information provided by 18 F-FDG/PET has strong prognostic utility in ESCC.^32^ The significant reduction in SUV max is correlated with the corresponding histopathological response^33^ and serve as a prognostic factor in the clinical efficacy of neoadjuvant chemoradiotherapy for patients with oesophageal cancer.^34,35^ The TRG was proposed as a new classification system to evaluate histologic tumour regression after chemoradiotherapy in ESCC.^36^ In the current study, we revealed the relationship between *F. nucleatum* and chemotherapeutic resistance in ESCC using all three evaluation methods (i.e., SUV max values and TRG). In addition, we also confirmed this correlation using pre-treatment biopsy specimens and in vitro assays. The presence of *F. nucleatum* in tumour lesions might directly affect the efficacy of chemotherapy and serve as a predictor of chemosensitivity in ESCC patients.

As an oncogenic microorganism, *F. nucleatum* interacts through its FAP2 protein with the host factor, Gal-GalNAc, on the surface of CRC cells to mediate fusobacterial enrichment in CRC tissues.^37^ Previous studies showed that a high concentration of *F. nucleatum* accelerates the proliferation of CRC cells and subsequent antibiotic treatment reverses this effect.^38–40^ Although we have previously reported that *F. nucleatum* in oesophageal cancer tissues is associated with reduced survival, the mechanism(s) by which it affects the oesophageal tumour behaviour remains unexplained. In this study, the combination of TEM and LSCM indicated that *F. nucleatum* bind to ESCC cells through adhesion and invasion. Moreover, we also found that *F. nucleatum* survived in the host cells for at least 4 h. To the best of our knowledge, this is the first study revealing the dynamic state of *F. nucleatum* in oesophageal cancer cells. Future study is warranted to unravel the mechanism by which *F. nucleatum* affects aggressive tumour phenotype.

Available literature sources indicate that cancer therapies induce autophagy. On the other hand, autophagy also contributed to therapeutic resistance as well. The mammalian target of rapamycin activity, cancer stem cell phenotype, DNA damage-induced p53 activity, reactive oxygen species, and turnover of FOXO3A might contribute to autophagy-mediated drug resistance in human cancers.^41^ An increasing number of studies provides rational evidence on combining cancer therapeutic approaches with autophagy inhibitors, including CQ and other targeted small molecule inhibitors for better therapeutic efficacy. Therefore, a better understanding of autophagy in drug resistance is vital to improve therapeutic outcomes. Intriguingly, in the past few years, breakthrough studies elucidate the role of intestinal microbiota on the patients’ response to treatment.^11,19,42^ Our study revealed that *F. nucleatum* is significantly related to the outcomes of preoperative treatment. The in vitro assay results provide an insight into the chemoresistance induced by *F nucleatum* to 5-FU, CDDP, and Docetaxel in ESCC cells. We also showed that *F. nucleatum* induces LC3-II expression and autophagosome formation in ESCC cells. We observed that *F. nucleatum* increases ATG7 and Beclin-1 in ESCC to promote autophagy. In addition, the chemoresistance induced by *F. nucleatum* was reversed by inhibiting autophagy. Thus we conclude that autophagy plays an important role in *F. nucleatum-*mediated chemoresistance to 5-FU, CDDP, and Docetaxel in ESCC cells. Our findings might shed light on the mechanism by which *F. nucleatum* affects chemoresistance in ESCC. Despite these promising results, to completely elucidate this mechanism, future studies including in vivo experiment are warranted to investigate the upstream regulatory mechanism of *F. nucleatum* and its chemoresistance mechanism on ESCC. The present investigation might potentially help in the emergence of *F. nucleatum* DNA as a prognostic biomarker.

Accumulating evidence suggests that intestinal microorganisms modulate the host response to chemotherapeutic and immunotherapeutic drugs. Hence, the gut microbiota is critical for the development of personalised cancer treatment strategies.^11,43–45^ Our present study further confirmed that *F. nucleatum* induces chemoresistance by autophagy induction in ESCC. Therefore, *F. nucleatum* may be a promising therapeutic target against ESCC and the combination of anti-*F. nucleatum* treatment in patients with a relatively high amount of *F. nucleatum* can improve the efficiency of chemotherapy. By extending the investigation on probing the mechanistic role of *F. nucleatum* in ESCC, it might contribute to designing better approaches for cancer diagnosis, prevention, and treatment.

## Supplementary information

supple tables and figures

## Data Availability

The data sets used and/or analysed during the current study are available from the corresponding author on reasonable request.

## References

[1] Pennathur A, Gibson MK, Jobe BA, Luketich JD (2013). Oesophageal carcinoma. Lancet.

[2] Siegel RL, Miller KD, Jemal A (2017). Cancer statistics, 2017. CA Cancer J. Clin..

[3] Siegel R, Ma J, Zou Z, Jemal A (2014). Cancer statistics, 2014. CA Cancer J. Clin..

[4] Sjoquist KM, Burmeister BH, Smithers BM, Zalcberg JR, Simes RJ, Barbour A (2011). Survival after neoadjuvant chemotherapy or chemoradiotherapy for resectable oesophageal carcinoma: an updated meta-analysis. Lancet Oncol..

[5] Ajani JA, D’Amico TA, Almhanna K, Bentrem DJ, Besh S, Chao J (2015). Esophageal and esophagogastric junction cancers, version 1.2015. J. Natl Compr. Canc Netw..

[6] Hara H, Tahara M, Daiko H, Kato K, Igaki H, Kadowaki S (2013). Phase II feasibility study of preoperative chemotherapy with docetaxel, cisplatin, and fluorouracil for esophageal squamous cell carcinoma. Cancer Sci..

[7] Ojima T, Nakamori M, Nakamura M, Katsuda M, Hayata K, Kato T (2016). Neoadjuvant chemotherapy with divided-dose docetaxel, cisplatin and fluorouracil for patients with squamous cell carcinoma of the esophagus. Anticancer Res..

[8] Panebianco C, Andriulli A, Pazienza V (2018). Pharmacomicrobiomics: exploiting the drug-microbiota interactions in anticancer therapies. Microbiome.

[9] Routy B, Le Chatelier E, Derosa L, Duong CPM, Alou MT, Daillere R (2018). Gut microbiome influences efficacy of PD-1-based immunotherapy against epithelial tumors. Science.

[10] Sivan A, Corrales L, Hubert N, Williams JB, Aquino-Michaels K, Earley ZM (2015). Commensal Bifidobacterium promotes antitumor immunity and facilitates anti-PD-L1 efficacy. Science.

[11] Geller LT, Barzily-Rokni M, Danino T, Jonas OH, Shental N, Nejman D (2017). Potential role of intratumor bacteria in mediating tumor resistance to the chemotherapeutic drug gemcitabine. Science.

[12] Kostic AD, Chun E, Robertson L, Glickman JN, Gallini CA, Michaud M (2013). *Fusobacterium nucleatum* potentiates intestinal tumorigenesis and modulates the tumor-immune microenvironment. Cell Host Microbe.

[13] McCoy AN, Araujo-Perez F, Azcarate-Peril A, Yeh JJ, Sandler RS, Keku TO (2013). Fusobacterium is associated with colorectal adenomas. PLoS ONE.

[14] Tahara T, Yamamoto E, Suzuki H, Maruyama R, Chung W, Garriga J (2014). Fusobacterium in colonic flora and molecular features of colorectal carcinoma. Cancer Res..

[15] Bui FQ, Johnson L, Roberts J, Hung SC, Lee J, Atanasova KR (2016). *Fusobacterium nucleatum* infection of gingival epithelial cells leads to NLRP3 inflammasome-dependent secretion of IL-1beta and the danger signals ASC and HMGB1. Cell Microbiol..

[16] Suehiro Y, Sakai K, Nishioka M, Hashimoto S, Takami T, Higaki S (2017). Highly sensitive stool DNA testing of *Fusobacterium nucleatum* as a marker for detection of colorectal tumours in a Japanese population. Ann. Clin. Biochem..

[17] Yamamura K, Baba Y, Nakagawa S, Mima K, Miyake K, Nakamura K (2016). Human microbiome *Fusobacterium Nucleatum* in esophageal cancer tissue is associated with prognosis. Clin. Cancer Res..

[18] Yamamura, K., Izumi, D., Kandimalla, R., Sonohara, F., Baba, Y., Yoshida, N. et al. Intratumoral *Fusobacterium nucleatum* levels predict therapeutic response to neoadjuvant chemotherapy in esophageal squamous cell carcinoma. *Clin. Cancer Res.* **25**, 6170–6179 (2019). 10.1158/1078-0432.CCR-19-0318 PMC6801075 31358543

[19] Yu T, Guo F, Yu Y, Sun T, Ma D, Han J (2017). *Fusobacterium nucleatum* promotes chemoresistance to colorectal cancer by modulating autophagy. Cell.

[20] Edge SB, Compton CC (2010). The American Joint Committee on Cancer: the 7th edition of the AJCC cancer staging manual and the future of TNM. Ann. Surg. Oncol..

[21] Izumi D, Yoshida N, Watanabe M, Shiraishi S, Ishimoto T, Kosumi K (2016). Tumor/normal esophagus ratio in (18)F-fluorodeoxyglucose positron emission tomography/computed tomography for response and prognosis stratification after neoadjuvant chemotherapy for esophageal squamous cell carcinoma. J. Gastroenterol..

[22] Chirieac LR, Swisher SG, Ajani JA, Komaki RR, Correa AM, Morris JS (2005). Posttherapy pathologic stage predicts survival in patients with esophageal carcinoma receiving preoperative chemoradiation. Cancer.

[23] Mima K, Sukawa Y, Nishihara R, Qian ZR, Yamauchi M, Inamura K (2015). *Fusobacterium nucleatum* and T cells in colorectal carcinoma. JAMA Oncol..

[24] Saito Y, Fujii R, Nakagawa KI, Kuramitsu HK, Okuda K, Ishihara K (2008). Stimulation of *Fusobacterium nucleatum* biofilm formation by Porphyromonas gingivalis. Oral. Microbiol. Immunol..

[25] Rubinstein MR, Wang X, Liu W, Hao Y, Cai G, Han YW (2013). *Fusobacterium nucleatum* promotes colorectal carcinogenesis by modulating E-cadherin/beta-catenin signaling via its FadA adhesin. Cell Host Microbe.

[26] Tanida I, Minematsu-Ikeguchi N, Ueno T, Kominami E (2005). Lysosomal turnover, but not a cellular level, of endogenous LC3 is a marker for autophagy. Autophagy.

[27] Tanida I, Ueno T, Kominami E (2008). LC3 and autophagy. Methods Mol. Biol..

[28] Mandelbaum J, Rollins N, Shah P, Bowman D, Lee JY, Tayber O (2015). Identification of a lung cancer cell line deficient in atg7-dependent autophagy. Autophagy.

[29] Kuma A, Matsui M, Mizushima N (2007). LC3, an autophagosome marker, can be incorporated into protein aggregates independent of autophagy: caution in the interpretation of LC3 localization. Autophagy.

[30] Backer JM (2008). The regulation and function of class III PI3Ks: novel roles for Vps34. Biochem. J..

[31] Aghdaei HA, Kadijani AA, Sorrentino D, Mirzaei A, Shahrokh S, Balaii H (2018). An increased Bax/Bcl-2 ratio in circulating inflammatory cells predicts primary response to infliximab in inflammatory bowel disease patients. U. Eur. Gastroenterol. J..

[32] Greally M, Chou JF, Molena D, Rusch VW, Bains MS, Park BJ (2018). Positron-emission tomography scan-directed chemoradiation for esophageal squamous cell carcinoma: no benefit for a change in chemotherapy in positron-emission tomography nonresponders. J. Thorac. Oncol..

[33] Sasaki K, Uchikado Y, Okumura H, Omoto I, Kita Y, Arigami T (2017). Role of (18)F-FDG-PET/CT in esophageal squamous cell carcinoma after neoadjuvant chemoradiotherapy. Anticancer Res..

[34] Beukinga RJ, Hulshoff JB, Mul VEM, Noordzij W, Kats-Ugurlu G, Slart R (2018). Prediction of response to neoadjuvant chemotherapy and radiation therapy with baseline and restaging (18)F-FDG PET imaging biomarkers in patients with esophageal cancer. Radiology.

[35] Huang YC, Lu HI, Huang SC, Hsu CC, Chiu NT, Wang YM (2017). FDG PET using SUVmax for preoperative T-staging of esophageal squamous cell carcinoma with and without neoadjuvant chemoradiotherapy. BMC Med. Imaging.

[36] Mandard AM, Dalibard F, Mandard JC, Marnay J, Henry-Amar M, Petiot JF (1994). Pathologic assessment of tumor regression after preoperative chemoradiotherapy of esophageal carcinoma. Clinicopathologic correlations. Cancer.

[37] Abed J, Emgard JE, Zamir G, Faroja M, Almogy G, Grenov A (2016). Fap2 mediates *Fusobacterium nucleatum* colorectal adenocarcinoma enrichment by binding to tumor-expressed Gal-GalNAc. Cell Host Microbe.

[38] Bullman S, Pedamallu CS, Sicinska E, Clancy TE, Zhang X, Cai D (2017). Analysis of Fusobacterium persistence and antibiotic response in colorectal cancer. Science.

[39] Castellarin M, Warren RL, Freeman JD, Dreolini L, Krzywinski M, Strauss J (2012). *Fusobacterium nucleatum* infection is prevalent in human colorectal carcinoma. Genome Res..

[40] Bashir A, Miskeen AY, Bhat A, Fazili KM, Ganai BA (2015). *Fusobacterium nucleatum*: an emerging bug in colorectal tumorigenesis. Eur. J. Cancer Prev..

[41] Smith AG, Macleod KF (2019). Autophagy, cancer stem cells and drug resistance. J. Pathol..

[42] Kroemer G, Zitvogel L (2018). Cancer immunotherapy in 2017: the breakthrough of the microbiota. Nat. Rev. Immunol..

[43] Alexander JL, Wilson ID, Teare J, Marchesi JR, Nicholson JK, Kinross JM (2017). Gut microbiota modulation of chemotherapy efficacy and toxicity. Nat. Rev. Gastroenterol. Hepatol..

[44] Vande Voorde J, Sabuncuoglu S, Noppen S, Hofer A, Ranjbarian F, Fieuws S (2014). Nucleoside-catabolizing enzymes in mycoplasma-infected tumor cell cultures compromise the cytostatic activity of the anticancer drug gemcitabine. J. Biol. Chem..

[45] Gui QF, Lu HF, Zhang CX, Xu ZR, Yang YH (2015). Well-balanced commensal microbiota contributes to anti-cancer response in a lung cancer mouse model. Genet. Mol. Res..

