# Supplementary material for: Fusobacterium nucleatum confers chemoresistance by modulating autophagy in oesophageal squamous cell carcinoma
Source: Br J Cancer. 2020 Dec 10;124(5):963–74. doi: 10.1038/s41416-020-01198-5 (PMC7921654; doi:10.1038/s41416-020-01198-5)
Supplement: Supplementary file 1 — supple tables and figures [file 41416_2020_1198_MOESM1_ESM.pdf]

Supplementary Table 1.

Clinicopathological features in 95 patients with PET data

	F. nucleatum expression		
	Low (n=68)	High (n=27)	*p-value*
Mean age	67	64	0.08
Sex			0.69
Male	60	26	
Female	8	1	
Location			0.54
Upper	22	7	
Lower	46	20	
T stage			0.12
T1	9	3	
T2	12	1	
T3-	47	23	
N stage			0.82
N0	6	2	
N1-3	62	25	

Supplementary Table 2.

Clinicopathological features of Cohort B

	Total N=30
Mean age	69
Sex	
Male	27
Female	3
Location	
Upper	11
Lower	19
Stage	
II	7
III	10
IV	13

Supplementary Table 3: cohort C

	ATG-7 expression		
	Low (n=225)	High (n=59)	*p-value*
Mean age ± SD	66 (41-89)	66 (48-80)	0.72
Sex			0.17
Male	202	49	
Female	23	10	
Performance status			0.88
0	173	47	
1	42	10	
2	10	2	
Tobacco use			0.18
yes	194	55	
no	31	4	
Alcohol use			1.0
yes	214	57	
no	11	2	
Stage			0.02
0	16	0	
I	65	10	
II	59	21	
III	58	20	
IV	26	8	
Preoperative therapy			0.54
present	87	20	
Absent	138	39	

Supplementary Figures

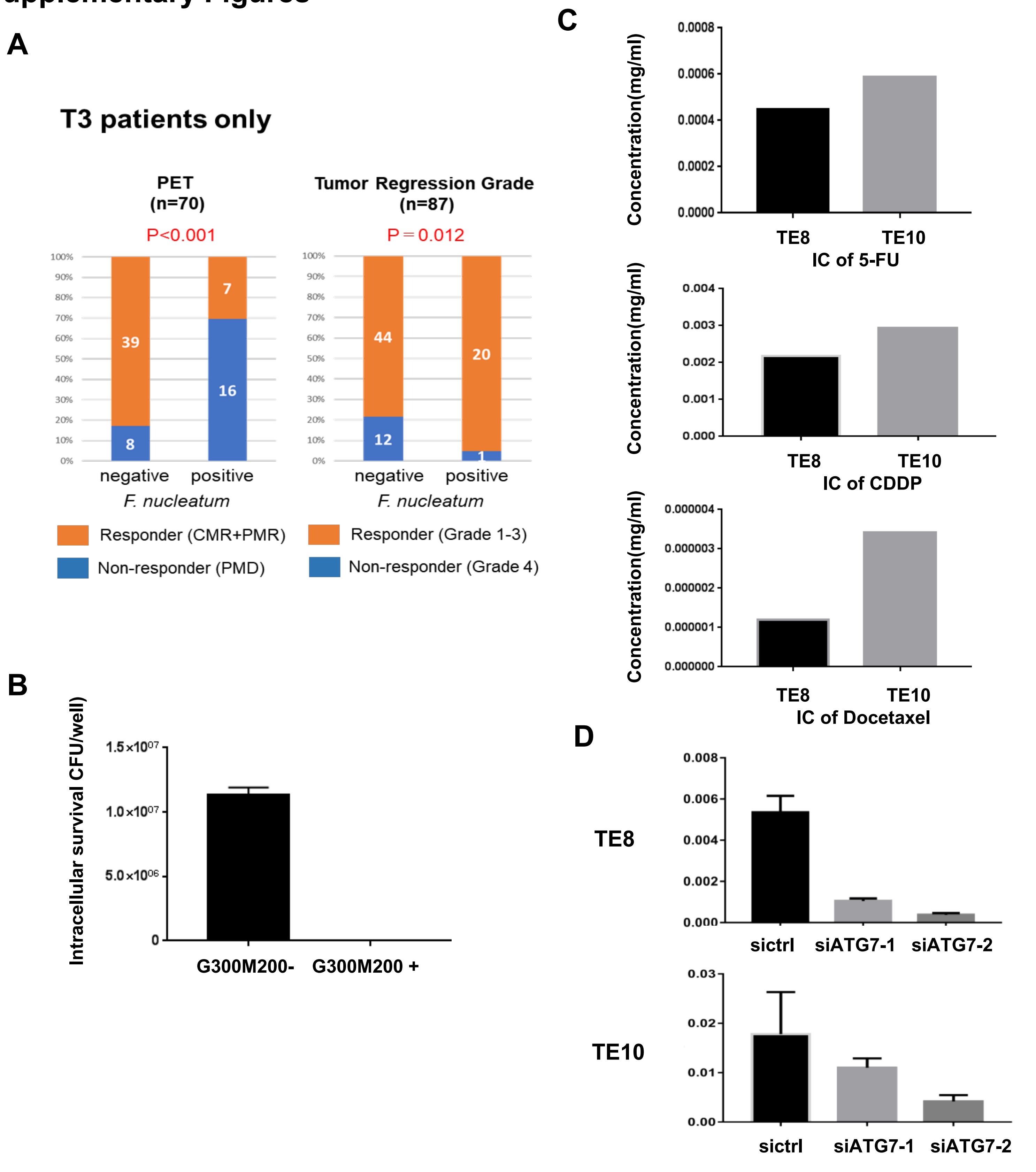

A. The responses of SUVmax and Tumor Regression Grade according to *F. nucle*atum status among T3 patients only in Cohort A**.**

B. Determination of minimum inhibitory concentration (MIC) to demonstrate that metronidazole (200µg/ml) and gentamicin (300µg/ml) kill 5X10^{7}/ml *F. nucleatum* in 1 h completely.

C. Half-maximal inhibitory concentration (IC50) of 5-FU, CDDP, and Docetaxel in TE8 and TE10 cells calculated according to growth assay.

D. qPCR analysis to check the mRNA levels of ATG7 upon knockdown using siATG7-1 and siATG7-2 in 72 hours.

E

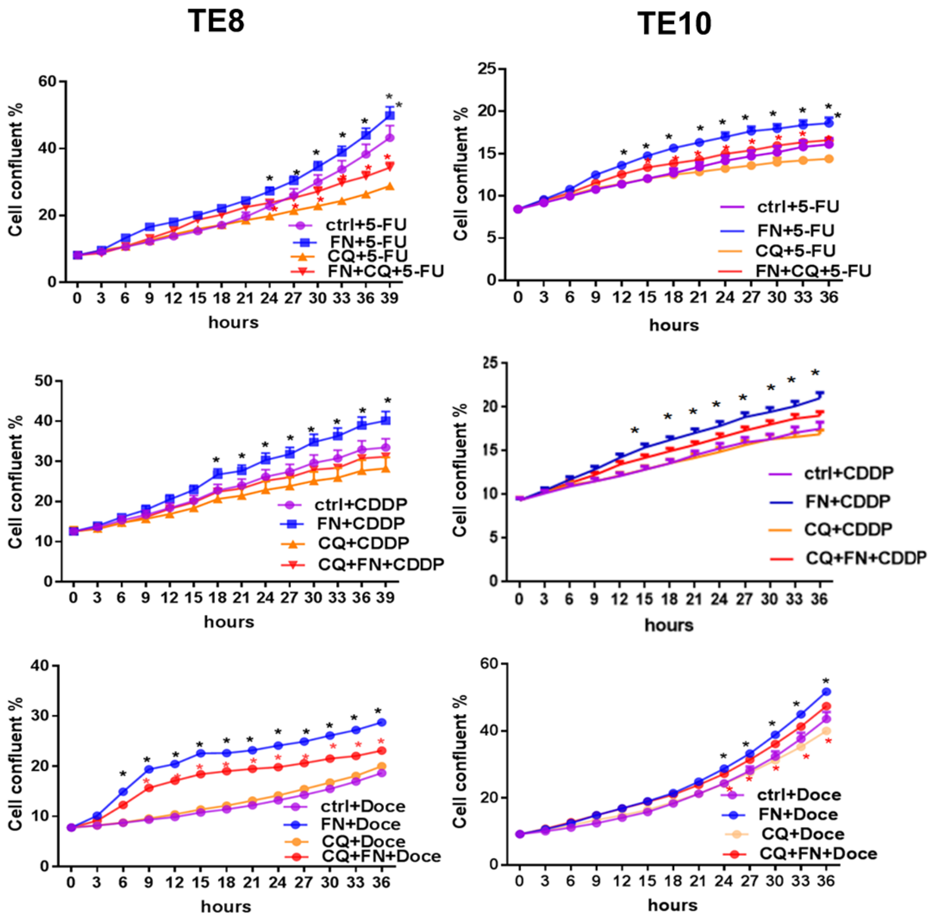

F

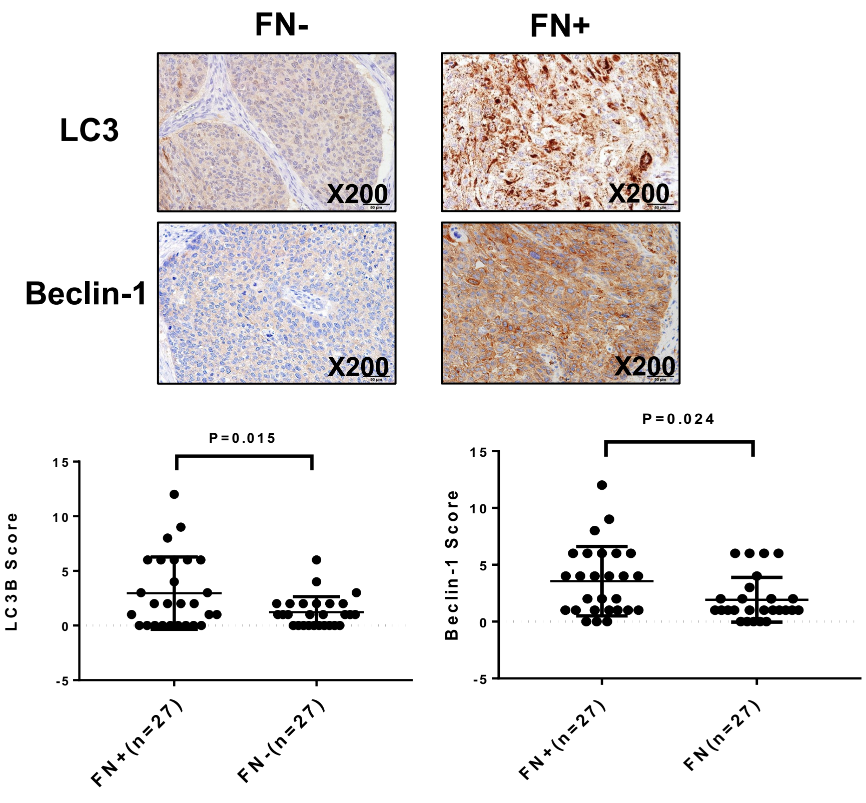

G

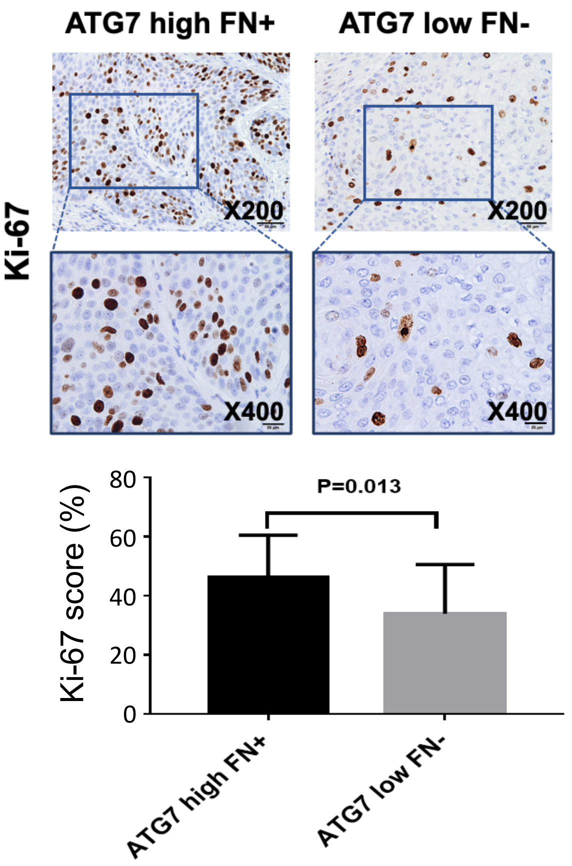

E. Cell proliferation assay in IncuCyte showing that CQ could decrease the growth of TE8 and TE10 in *F. nucleatum* group compared with that in only *F. nucleatum* group. * P<0.05 (red and black) indicate significant difference between ctrl and *F. nucleatum* groups and CQ and CQ+ *F. nucleatum* groups.

F. Immunohistochemical analysis of LC3 and Beclin-1protein expression tumor lesions from ESCC patients. Higher LC3 and Beclin-1 score were observed in the *F. nucleatum* (+) group compared to the *F. nucleatum* (-) group (P=0.015 and P=0.024, respectively)

G. Immunohistochemical analysis of Ki-67 expression from ESCC patients' sample. Higher Ki-67 were observed in the ATG7 high *F. nucleatum* (+) group compared to the ATG7 low *F. nucleatum* (-) group (P=0.013).

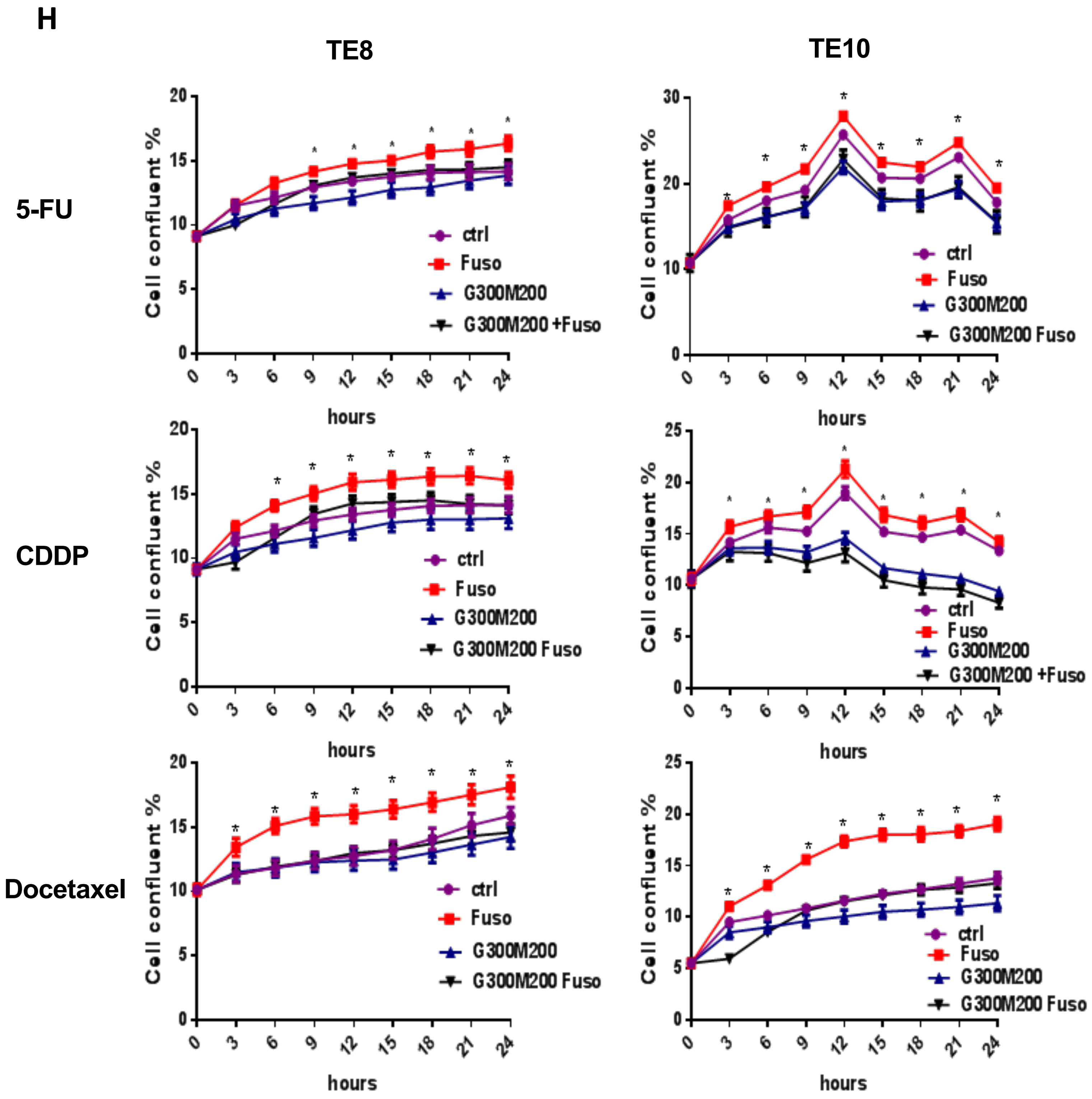

H. Cell proliferation assay in IncuCyte showing that gentamicin (300 μg/ml) and metronidazole (200 μg/ml) could decrease the growth of TE8 and TE10 in *F. nucleatum* group treated with 5-FU, CDDP, and Docetaxel. * P<0.05 indicate significant difference between *F. nucleatum* group and *F. nucleatum* treated with gentamicin plus metronidazole group.
